# Pediatric Autoimmune Sclerosing Cholangitis: Diagnostic and Therapeutic Challenges

**DOI:** 10.3390/pediatric18020054

**Published:** 2026-04-08

**Authors:** Raisa-Maria Sucaciu, Alina Grama, Alexandra Mititelu, Bianca Raluca Mariș, Ioana Filimon, Bobe Petrushev, Daniel Cristian Popescu, Gabriel Benţa, Tudor Lucian Pop

**Affiliations:** 1 2nd Pediatric Discipline, Department of Mother and Child, “Iuliu Hațieganu” University of Medicine and Pharmacy, 400012 Cluj-Napoca, Romania; sucaciu.raisamaria@elearn.umfcluj.ro (R.-M.S.); perta.alexandra@elearn.umfcluj.ro (A.M.); mateescu_bianca_raluca@elearn.umfcluj.ro (B.R.M.); popescu.danielcristian@elearn.umfcluj.ro (D.C.P.); benta.gabriel@elearn.umfcluj.ro (G.B.); tudor.pop@umfcluj.ro (T.L.P.); 2 2nd Pediatric Clinic, Emergency Clinical Hospital for Children, 400217 Cluj-Napoca, Romania; 3 Radiology Department, Children’s Emergency Clinical Hospital, 400378 Cluj-Napoca, Romania; ifilimon@yahoo.com; 4 Department of Pathology, “Octavian Fodor” Regional Institute of Gastroenterology and Hepatology, 400162 Cluj-Napoca, Romania; bobe.petrushev@gmail.com

**Keywords:** autoimmune hepatitis, sclerosing cholangitis, liver fibrosis, ulcerative colitis, ulcerative stomatitis

## Abstract

Background. Autoimmune sclerosing cholangitis (ASC) is a rare clinical entity characterized by overlapping features of autoimmune hepatitis and primary sclerosing cholangitis. It predominantly affects pediatric patients. Therapeutic management is often complex, requiring a multidisciplinary and individualized approach, especially in the context of associated autoimmune diseases. Case presentation. We present the case of a female patient diagnosed at the age of 10 with ASC, for which immunosuppressive therapy with prednisone, azathioprine (AZA), and ursodeoxycholic acid (UDCA) was initiated, with an initially favorable course. One year later, following a Severe Acute Respiratory Syndrome Coronavirus-2 (SARS-CoV-2) infection, the patient experienced reactivation of liver disease and subsequently developed ulcerative pancolitis (UC), for which 5-aminosalicylic acid (5-ASA) therapy was initiated. Due to repeated hepatic flares and/or colitis relapses, therapy was escalated successively to mycophenolate mofetil, tacrolimus, and eventually infliximab (IFX). Despite treatment, the liver disease progressed, culminating in liver cirrhosis. Our patient developed portal hypertension and esophageal varices, with two episodes of upper gastrointestinal bleeding requiring endoscopic band ligation. At the age of 14, the patient developed recurrent episodes of non-infectious ulcerative stomatitis. Biopsy of the lesions revealed non-specific chronic inflammation, unrelated to colitis activity (confirmed microscopic remission of UC). By exclusion, an adverse drug reaction was suspected, with AZA being the most likely cause. Following its discontinuation, the lesions resolved. Beyond the physiological and therapeutic aspects, the patient displays marked emotional fragility due to prolonged and repeated hospitalizations (18 out of 60 months), which have impacted treatment adherence. Conclusions. This case highlights the complexity of managing pediatric patients with multiple autoimmune diseases. The necessary combination of immunosuppressive therapies may lead to significant adverse effects and further complicate disease progression. Moreover, psychological components play a crucial role in treatment compliance and therapeutic success, emphasizing the need for an integrated approach that includes specialized psychological support.

## 1. Introduction

Autoimmune sclerosing cholangitis (ASC) is a rare clinical entity characterized by overlapping features of autoimmune hepatitis (AIH) and primary sclerosing cholangitis (PSC). It is defined by inflammation, dilation, narrowing and obliteration of the bile ducts, both intrahepatic and extrahepatic, visible on magnetic resonance cholangiography (MRC) or histopathologically. The patients present with elevated serum globulin levels, the presence of antinuclear (ANA) and/or anti-smooth muscle antibodies (SMA) or circulating positive perinuclear anti-neutrophil cytoplasmic antibodies (pANCA) and inflammatory changes on liver histology, leading to the destruction of liver parenchyma and progression to fibrosis and cirrhosis. It predominantly affects pediatric patients, regardless of age or gender [1].

Other features suggestive of biliary disease are elevated cholestatic enzymes, large splenomegaly, portal hypertension, and evidence of inflammatory bowel disease (IBD), present in 40% of patients [1,2].

All patients with AIH and cholestasis should be evaluated with MRC to assess for underlying biliary disease and to support the diagnosis of ASC. The course of treatment and the prognosis are different than the evolution of AIH. Patients with ASC require ursodeoxycholic acid in addition to immunosuppressive medication (prednisone) alone or in combination with azathioprine (AZA) as first-line treatment, and tend to have a more aggressive and treatment-resistant course of disease, leading more often to liver transplantation [1,2].

Therapeutic management is often complex, requiring a multidisciplinary, individualized approach, especially in the context of other autoimmune diseases.

This report aims to present the case of a teenage patient diagnosed with three autoimmune diseases and the challenges it brought in both therapeutic and emotional management.

## 2. Case Presentation

We present the case of a girl diagnosed at 10 years old with ASC, for which immunosuppressive therapy with prednisone, AZA, and ursodeoxycholic acid (UDCA) was initiated with an initially favorable course, but with further challenges in the disease evolution and therapies.

**Figure 1:**
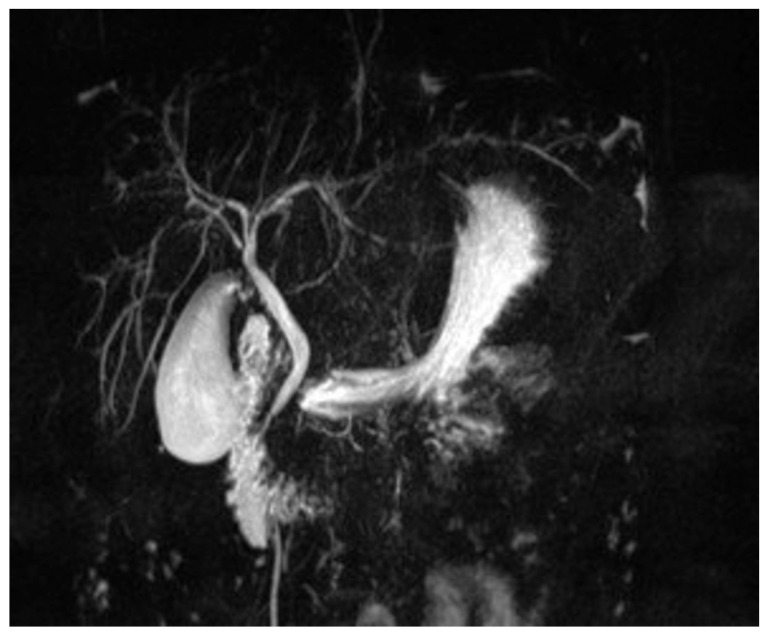
MRC imaging of our patient, showing minimal dilation of the intrahepatic bile ducts.

**Figure 2:**
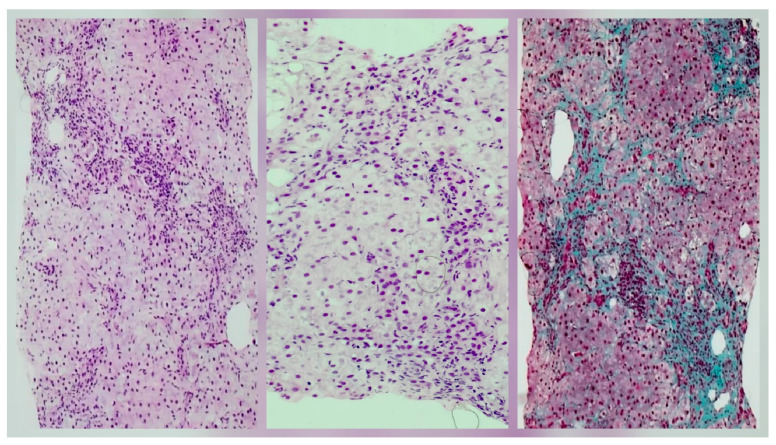
Histology of hepatic tissue; the first two pictures show hematoxylin–eosin (H and E) stain. Hepatic parenchyma with portal tracts showing inflammatory infiltrates composed predominantly of lymphocytes, with rare plasma cells and occasional neutrophils, with ductular reaction phenomena and lymphoepithelial lesions. The last picture shows a special stain, Masson’s trichrome, which highlights fibrosis at the level of the portal tracts, with fibrous septal extensions into acinar zone one, as well as areas of pericellular/perisinusoidal fibrosis. The fibrosis stage is between F1 and F2.

The patient was first referred to our clinic in June 2020, at the age of ten, for fatigue, loss of appetite, jaundice, and dark urine. On the clinical exam, the patient presented jaundice, hepatomegaly (3 cm below the ribs), and reduced fatty subcutaneous tissue. Laboratory findings included elevated transaminases (aspartate aminotransferase [AST] 156 U/L, alanine aminotransferase [ALT] 124 U/L), cholestasis (alkaline phosphatase [ALP] 550 U/L and gamma-glutamyl transferase [GGT] 435 U/L), increased total bilirubin (TB) 6.46 mg/dL and direct bilirubin (DB) 4.02 mg/dL, elevated immunoglobulin G (IgG) levels (5293 mg/dL), and with positive circulating auto-antibodies, ANA (1/320) and pANCA. She also presented autoimmune thyroiditis (positive anti-thyroid peroxidase, anti-TPO, antibodies), but thyroid function was normal. The MRC detected minimal dilation of the intrahepatic bile ducts (Figure 1) and the liver biopsy described interface hepatitis with inflammation and bridging fibrosis, indicating a fibrosis score between F1 and F2 (mild to moderate fibrosis) (Figure 2).

All these clinical, laboratory, imaging, and histological findings support the diagnosis of ASC. We started immunosuppressive and choleretic treatment, according to the European Society for Paediatric Gastroenterology, Hepatology and Nutrition (ESPGHAN) 2019 Guide [3], with prednisone (1 mg/kg/day with gradual tapering), AZA (1 mg/kg/day initially and up to 2.5 mg/kg/day) and UDCA (15 mg/kg/day).

The evolution was favorable, with both clinical and biological remission (normal transaminases, GGT and ALP, IgG levels, and decreased ANA titre (1/20)). One year later, following a SARS-CoV-2 infection, the patient experienced reactivation of her liver disease, with no response to the increase in first-line immunosuppressants. Second-line treatment with mycophenolate mofetil (MMF-20 mg/kg/day) was started in order to induce and maintain remission.

**Figure 3:**
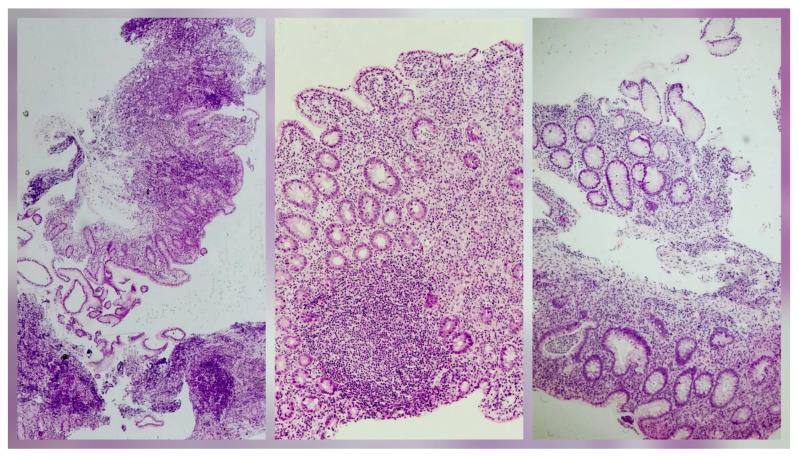
Histology of the colonic tissue, showing hematoxylin–eosin (H and E) stain. Colonic mucosa with erosions and architectural distortion (crypts showing loss of parallel alignment and irregular contours, some with a branching tendency). Neutrophilic activity is present (cryptitis and crypt abscesses). The epithelium shows reactive changes.

In May 2022, she was again admitted to our hospital, with bloody stools and abdominal pain, associated with an important inflammatory syndrome (elevated CRP and ESR), elevated transaminases and IgG levels, and positive pANCA. No infectious agent was found, including *Clostridioides difficile*, and a bowel ultrasound showed submucosal thickening of the colon. The colonoscopy with tissue sampling and histopathological examination indicated ulcerative colitis (UC) with backwash ileitis (Figure 3). Derivatives of 5-aminosalicylic acid (5-ASA) were subsequently added to the treatment plan, with clinical and biological remission.

During this time, liver disease progressed, and the patient developed portal hypertension with splenomegaly and esophageal varices, complicated with two episodes of upper gastrointestinal bleeding that required endoscopic band ligation in January and February 2023. According to guidelines, we changed immunosuppressive therapy to tacrolimus (0.05 mg/kg/day) given the poor treatment response, the progression of the liver disease, and the association between MMF and 5-ASA derivatives, which could potentially increase the risk of variceal bleeding. Later, she developed two episodes of *Clostridioides difficile* infection, treated with oral vancomycin.

Due to repeated hepatic flares and inflammatory bowel disease (IBD) relapses, therapy was escalated to infliximab (IFX-5 mg/kg/dose) in June 2023. After what seemed to be a period of stability, with good treatment response, we decided to reinitiate treatment with AZA (2.5 mg/kg/day) and prednisone (5 mg/day), as maintenance therapy, along with periodic IFX infusions.

**Figure 4:**
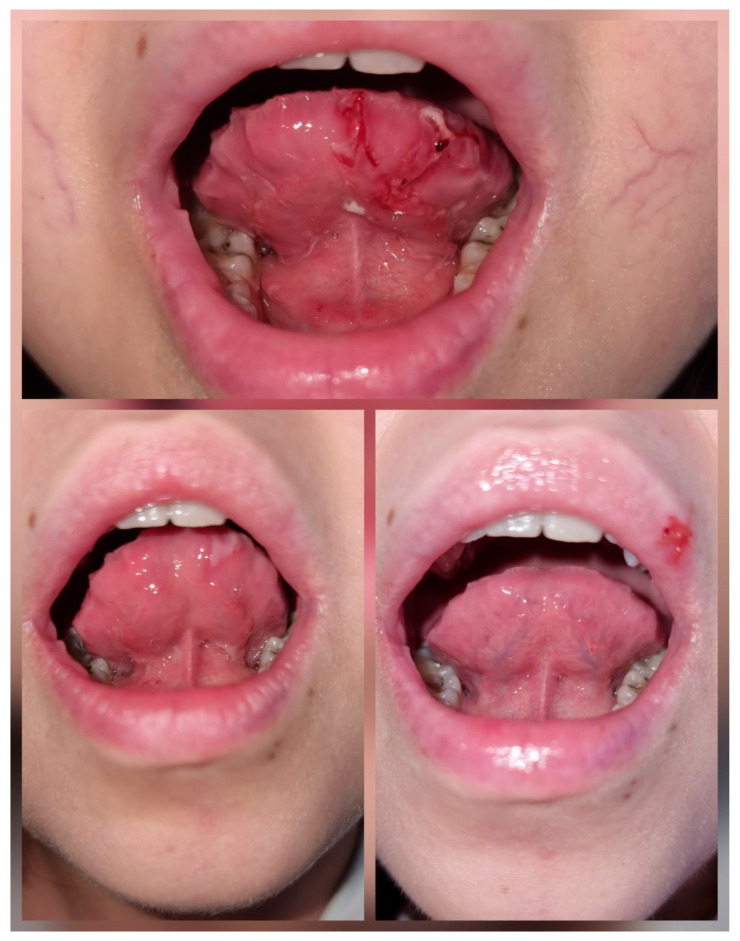
Oral ulcerations during azathioprine treatment (**top**), one week (**bottom left**) and six weeks (**bottom right**) after its discontinuation.

**Figure 5:**
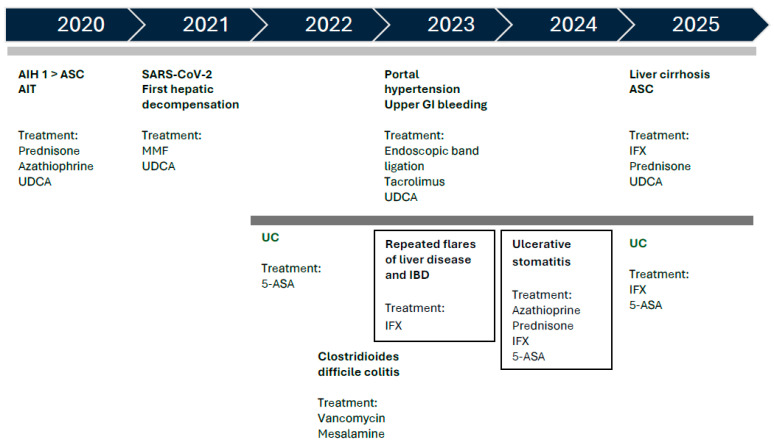
Timeline of disease evolution and therapy. (AIH, autoimmune hepatitis; ASC, autoimmune sclerosing cholangitis; AIT, autoimmune thyroiditis; UDCA, ursodeoxycholic acid; SARS-CoV-2, Severe Acute Respiratory Syndrome Coronavirus-2; MMF, mycophenolate mofetil; GI, gastrointestinal; IFX, infliximab; UC, ulcerative colitis; 5-ASA, 5-aminosalicylic acid; IBD, inflammatory bowel disease.)

At the age of 14, in December 2024, the patient developed recurrent episodes of ulcerative stomatitis (Figure 4). We first excluded infectious causes, herpes simplex virus [HSV-1, HSV-2, HSV-6], Epstein–Barr virus [EBV], cytomegalovirus [CMV], toxoplasma, and human immunodeficiency virus [HIV]. A possible association with UC flare-ups was considered but was not supported by endoscopic appearance and histopathological findings. A lesional biopsy revealed features of chronic inflammation, allowing us to rule out a vascular etiology. By exclusion, an adverse drug reaction was suspected, with AZA being the most likely cause. We administered topical anti-inflammatory and pro-epithelialization treatment with vitamin A and local hemostatic agents, such as tranexamic acid and tannic acid solutions, the latter being the most effective for bleeding control. Following the discontinuation of AZA, the lesions resolved. Currently, the patient is receiving prednisone (5 mg/day), UDCA (20 mg/kg/day), IFX (6 mg/kg/dose), and 5-ASA (3.5 g/day), with favorable therapeutic management to date (Figure 5).

Considering the association of all these severe autoimmunities, we genetically tested our patient with a next-generation sequencing panel (Comprehensive Immune and Cytopenia panel, Blueprint Genetics, Espoo, Finland), the girl being heterozygous for a mutation in the regulator of telomere elongation helicase 1 gene (*RTEL1 c.3631_3634 del*, p.[Gln1211Glyfs*57], frameshift variant), which is likely pathogenic. This mutation is known to be associated with a later onset of myelodysplasia and liver disease [4], but it has not influenced her therapeutic management so far.

We also evaluated the Human Leukocyte Antigen (HLA) class II complex, the patient being positive for *HLA-DR6 (DR13)* and *HLA-DR1404*, corresponding to alleles DRB*13 and DRB*14.

Beyond the physiological and therapeutic aspects, the patient displays marked emotional fragility due to prolonged and repeated hospitalizations (18 out of 60 months), which have impacted treatment adherence.

## 3. Discussions

Autoimmune liver disorders (AILD) are traditionally categorized into three main clinical syndromes: AIH, a corticosteroid-responsive, relapsing-remitting hepatitis; PSC, a cholangitis affecting medium-to-large bile ducts with sclerosing features; and primary biliary cholangitis (PBC), a lymphocytic, granulomatous cholangitis involving small bile ducts [5].

Age at presentation plays a significant role in understanding these diseases. Notably, PBC is rarely seen in childhood, whereas AIH, PSC, and their overlapping variants are more common in pediatric populations. Clinicians often encounter patients, particularly younger ones, with overlapping biochemical, serological, and histological features of both PSC and AIH. This was frequently referred to as “PSC/AIH overlap”. In recent years, the term ASC has been used for this overlapping syndrome, which some suggest may be a distinct entity. However, current thinking increasingly views ASC not as a separate disease, but rather as an inflammatory phase of PSC that tends to occur earlier in the disease course, especially among younger individuals [5]. Our patient was diagnosed at the age of ten, which corresponds to the age pattern that this pathology follows.

The pathogenesis of AILD in children is unknown, but it is thought to involve complex interactions among environmental, genetic, and immune factors. Viruses, drugs, herbs and immunizations are among the triggers identified in this process. Infections with hepatitis viruses, herpes simplex virus (HSV-1, HSV-2, HSV-6), varicella-zoster virus (VZV), CMV, EBV, COVID-19, or measles may trigger immune-mediated destruction of hepatocytes and cholangiocytes [6]. In our case, the patient experienced a past EBV infection, detected through positive serology (IgG levels above 750 U/mL), but no clear trigger could be determined. One year following our patient’s diagnosis, a SARS-CoV-2 infection determined the reactivation of her liver disease, requiring immunosuppressive therapy escalation. Studies indicate that SARS-CoV-2 infection is associated with abnormal elevations of hepatic injury markers through multiple mechanisms, including direct hepatocellular injury, an exaggerated systemic inflammatory response, and hypoxic or ischaemic damage compounded by endothelial dysfunction. These processes may contribute to disease progression and increase the risk of decompensation in patients with AILD [7].

Polymorphisms in HLA alleles confer genetic predisposition to AILD. Different patterns have been observed across continents, with a higher incidence of DRB1*03:01, DRB1*04:01, and DRB3*01:01, which encode *HLA-DR3*, *HLA-DR4*, and *HLA-DR52*, respectively, in European and North American Caucasian populations. In the Japanese population, susceptibility was associated with the alleles DRB1*04:05, DRB1*04:01, and DQB1*04:01, as well as the heterozygous genotype DR4/DR8. In Argentina and Venezuela, the primary association with DRB1*13:01 was observed in children, and in Brazil, across all age groups [8]. A study from 2021 that aimed to investigate how the HLA profile can predict the severity of AILD in children of European ancestry found that the frequency of DRB1*13 in DRB1*03-negative patients was higher in patients with ASC [9]. The possession of DRB1*13 suggests a higher risk for developing ASC. At the same time, DRB1*03 and DRB1*07 were associated with AIH type 1 and type 2, respectively [9]. The influence of HLA class II genes on disease severity is strong: DRB1*03 homozygosity and DRB1*13 are associated with histologically more advanced disease from onset, while DRB1*07 is linked to the least optimal response to immunosuppression [10,11]. Our patient presented an HLA profile positive against *HLA-DR 6 (DR 13)* and *DR 1404*, with corresponding alleles DRB1*13 and DRB1*14. These results are consistent with findings reported in the medical literature. For the DRB1*14 allele, only a few cases have been reported in the literature. A study conducted in India found that DRB1*14 was independently associated with treatment response and predictive of difficult-to-treat disease [12]. In our case, the diagnosis of ASC was established at the age of 10 and the patient had a complicated course of the disease, with multiple reactivations, other autoimmune pathologies associated, and the necessity of permanently adjusting the treatment plan. Her HLA profile could explain the rather complicated course of treatment and the necessity to escalate medication lines. Another interesting aspect of our patient’s case is her unusual HLA profile; in Europe, patients typically have a higher incidence of DRB1*03:01, DRB1*04:01, and DRB3*01:01 [8].

The diagnosis of ASC can be established following specific algorithms. If a patient presents with increased transaminases and IgG levels, with specific autoantibodies (ANA or SMA, typical of type 1 AIH) and cholestasis (elevated ALP and GGT), pANCA should also be determined. If positive, the patient should undergo a MRC to assess the structural integrity of the biliary tract [2]. The “beaded” appearance of the bile ducts, due to multifocal strictures and dilations, with ductal wall thickening of the intrahepatic or extrahepatic biliary systems, is specific to ASC [13]. Liver biopsy can help document specific histological hallmarks for ASC, combining AIH and PSC features, and will establish the severity of the disease, but it can be rather non-specific in some cases, thus requiring correlation with MRC findings [2]. The pathology of ASC is complex. Typically, in AIH, hepatocytes are the primary targets of injury, with inflammation extending from the portal tract into adjacent lobules, forming an interface hepatitis. It can further progress to zone three necrosis or bridging necrosis. PSC is characterized by chronic inflammation and fibrosis of the bile ducts, affecting both intrahepatic and extrahepatic bile ducts. A hallmark feature is the concentric “onionskin” fibrosis that surrounds the affected ducts, progressively narrowing them, causing atrophy and eventually obstruction [14,15]. ASC combines these histopathological findings, as in the case of our patient’s liver biopsy result, showing all the characteristics described above, concluding the presence of autoimmune sclerosing cholangitis. Our patient had elevated liver enzymes and elevated IgG levels, along with positive ANA and pANCA. Although the MRC showed little evidence of bile duct disease, the histopathological findings described advanced fibrosis and sclerosing cholangitis.

The association between AILD and IBD is well known (up to 45%) [14]. AILD is mostly associated with UC (80%), but also with Crohn’s disease (20%) and other indeterminate forms of IBD. These comorbid conditions may impact the overall prognosis of the underlying liver disease [16]. Data indicate that patients with IBD and concurrent ASC are at increased risk for treatment-refractory disease, often necessitating early escalation and a more aggressive initial therapeutic approach [17]. All patients with ASC should undergo IBD screening with fecal calprotectin testing [5]. In our case, the patient was first diagnosed with ASC, and two years later, she developed UC. Multiple relapses of both hepatic and intestinal diseases were present, with the necessity to escalate treatment options to biological therapy with IFX, thus following the treatment-resistant pattern described in patients with concomitant ASC and IBD.

Management options of such challenging cases are complex. Adequate immunosuppression should be provided for both hepatic and intestinal pathologies. First-line induction therapy for ASC typically includes prednisone alone or combined with AZA, with combination therapy preferred due to fewer side effects, and also UDCA. Prednisone should be initiated at 0.5–1 mg/kg/day depending on disease severity. AZA (1–2 mg/kg/day) may be added, ideally 2 weeks after starting corticosteroids and when bilirubin is less than 6 mg/dL. MMF (1.5–2 g/day) has recently been considered as an alternative first-line option [2].

Medical management of pediatric UC includes 5-ASA, corticosteroids, thiopurines, and anti-TNF agents. Oral 5-ASA is the recommended first-line treatment for inducing and maintaining remission. Oral corticosteroids serve as second-line therapy for those unresponsive to 5-ASA, but intravenous corticosteroids remain the standard for severe UC. For long-term maintenance, thiopurines are recommended in children who are corticosteroid-dependent or experience frequent relapses. IFX should be considered for chronically active or steroid-dependent UC, uncontrolled by 5-ASA and thiopurines, for both induction and maintenance of remission [18].

Treatment options are, therefore, complex and should be individualized. In patients with concomitant diseases, it is important to balance and supplement therapy. It is necessary to monitor these patients frequently, as medication associations could also be responsible for adverse side effects. Our patient developed recurrent episodes of ulcerative stomatitis. We excluded infectious causes (HSV-1, HSV-2, HSV-6, EBV, CMV, Toxoplasma, and human immunodeficiency virus [HIV]) and a possible association with UC flare-ups (endoscopic appearance and histopathological findings from biopsies were normal, without any sign of IBD relapse). Lesional biopsy revealed features of chronic inflammation, allowing us to exclude a vascular etiology such as small-vessel vasculitis. We concluded that the lesions occurred as a result of chronic exposure to immunosuppressive medication, IFX or AZA, which are known to sometimes cause such ulcerations. Literature reports have described mucositis-type adverse reactions associated with AZA, as well as enhanced mucosal and bone marrow toxicity due to its interactions with 5-ASA derivatives [19]. Spacing out IFX infusions did not lead to any clinical improvement. Our patient also presented with worsening pancytopenia, which led us to discontinue AZA, resulting in the complete resolution of the ulcerative stomatitis.

Despite adequate treatment, patients with AILD are at increased risk of developing multiple complications. This can be further categorized into complications secondary to disease outcome (liver cirrhosis and portal hypertension), secondary to other associated pathologies (UC), and secondary to treatment. Our patient experienced a various number of complications (Table 1): she was first diagnosed with ASC, which, despite adequate treatment, progressed, culminating in liver cirrhosis. The patient developed portal hypertension with hypersplenism and esophageal varices, with two episodes of upper gastrointestinal bleeding requiring endoscopic band ligation. Two years later, following an episode of lower gastrointestinal bleeding, the diagnosis of UC was added. She experienced nutritional deficiencies, with delayed growth and development, mild osteoporosis, and hormonal imbalance, with amenorrhea and delayed puberty. The UC also progressed and was complicated by two episodes of *Clostridioides difficile* colitis (treated with oral vancomycin). The prolonged treatment with corticosteroids led to Cushing’s syndrome. The diverse treatment association was also responsible for promoting upper gastrointestinal bleeding (MMF and 5-ASA). At the age of 14, the patient developed recurrent episodes of ulcerative stomatitis, which was most probably caused by AZA treatment, also responsible for the persistence and aggravation of her cytopenia (also secondary to the hypersplenism).

Patients with multiple coexisting autoimmune disorders may experience a complex disease course. Long-term prognosis is influenced by the response to therapy, the progression and complications of the underlying conditions, potential drug interactions, and psychological factors. As AILD progresses to liver cirrhosis, the chronic hepatic insufficiency may lead to the necessity of liver transplantation. Few studies follow patients with pediatric onset AILD who have transitioned to adult care. Some studies suggest that one in three patients developed at least one liver-related adverse event, such as portal hypertension or the need for liver transplantation (LT), within two years of diagnosis [20]. Patients with ASC were found to be less responsive to treatment and to require further LT. Some studies also suggest that patients with ASC have a higher rate of liver complications, leading to mortality and the necessity of LT [21]. One of these complications is the development of hepatocellular carcinoma (HCC), with patients with ASC, cirrhosis, obesity, and high alcohol consumption being at higher risk [22]. Patients with associated ASC-IBD can also develop colorectal cancer, although at a lower prevalence [23]. The case of our patient raises a number of different questions. As detailed earlier, she developed numerous complications and was in need of multiple immunosuppressive treatments, with the presence of numerous adverse effects. We have currently tried all medication options available for children with ASC-IBD. In case of further liver and/or IBD decompensations, limited options remain available. She is at risk of developing HCC or colorectal cancer and may require LT as a last resort for ASC. The transition to adult care services may open some new therapeutic windows, with potential use of other immunosuppressants for the treatment of IBD, which could have positive effects on her liver disease [24].

During this time, the patient spent an overwhelming period of time hospitalized (18 out of 60 months), leading to psychological and emotional difficulties, with poor educational outcomes and low self-esteem, culminating in mild depression and anxiety. Beyond the physiological and therapeutic aspects of the complex management of patients with multiple autoimmune diseases, it is important not to neglect the psychological implications. Our patient displays marked emotional fragility due to prolonged and repeated hospitalizations, which have impacted treatment adherence but also her ability to adapt to her school environment, leading to the choice of online education. She is also experiencing adolescence, an added exacerbative factor to emotional instability, low self-esteem, and isolation, which can result in depression and anxiety. She presents a lower quality of life and lower treatment adherence. Patients should benefit from specialized psychological support, along with proper treatment and transition plans. The transition to adult healthcare services should start earlier, in order to give patients time to adjust to new changes, without creating further anxiety.

## 4. Conclusions

This case highlights the complexity of managing pediatric patients with multiple autoimmune diseases. The complexity of these challenging pathologies requires a combination of therapeutic approaches, from symptomatic treatment to immunosuppressive medication. This may lead to significant adverse effects and many possible drug interactions, which can further complicate the progression of the pathology. Moreover, the psychological component plays a crucial role in treatment compliance and therapeutic success, emphasizing the need for an integrated approach that includes specialized psychological support.

## Figures and Tables

**Table 1:** Overview of the patient’s complications.

ASC Complications	UC Complications	Treatment Complications
Liver cirrhosis	Systemic Infections (EBV, recurrent oral HSV, oral candidosis)	
Portal hypertension	Extraintestinal manifestations	Cushing’s syndrome
Esophageal varices with upper GI bleeding (two episodes)	Lower GI bleeding	Upper GI bleeding
Cytopenia due to hypersplenism	*Clostridioides difficile* colitis	Ulcerative stomatitis
Nutritional deficiencies (delayed growth and development) Hormonal disorders (amenorrhea, delayed puberty) Osteoporosis Prolonged hospitalization (18 out of the last 60 months) Psychological and emotional difficulties (poor educational outcomes, low self-esteem, mild depression and anxiety)		

ASC, autoimmune sclerosing cholangitis; UC, ulcerative colitis; EBV, Epstein–Barr virus; HSV, herpes simplex virus; GI, gastrointestinal.

## Data Availability

The original contributions presented in this study are included in the article. Further inquiries can be directed to the corresponding author.

## Abbreviations

The following abbreviations are used in this manuscript:

5-ASA	5-aminosalicylic acid
AIH	Autoimmune Hepatitis
AILD	Autoimmune Liver Diseases
AIT	Autoimmune Thyroiditis
ALP	Alkaline Phosphatase
ALT	Alanine Aminotransferase
ANA	Antinuclear Antibodies
ASC	Autoimmune Sclerosing Cholangitis
AST	Aspartate Aminotransferase
AZA	Azathioprine
CMV	Cytomegalovirus
DB	Direct Bilirubin
EBV	Epstein–Barr virus
ESPGHAN	European Society for Paediatric Gastroenterology, Hepatology and Nutrition
ESR	Erythrocyte Sedimentation Rate
GGT	Gamma-Glutamyl Transferase
HIV	Human Immunodeficiency Virus
HLA	Human Leukocyte Antigen
HSV	Herpes Simplex Virus
IBD	Inflammatory Bowel Disease
IFX	Infliximab
IgG	Immunoglobulin G
LT	Liver Transplantation
MMF	Mycophenolate Mofetil
MRC	Magnetic Resonance Cholangiography
pANCA	Perinuclear Anti-Neutrophil Cytoplasmic Antibodies
PBC	Primary Biliary Cholangitis
RTEL1	Regulator Of Telomere Elongation Helicase 1
SARS-CoV-2	Severe Acute Respiratory Syndrome Coronavirus-2
SMA	Smooth Muscle Antibodies
TB	Total Bilirubin
TNF	Tumor Necrosis Factor
TPO	Anti-Thyroid Peroxidase Antibodies
UC	Ulcerative Colitis
UDCA	Ursodeoxycholic Acid
VZV	Varicella-Zoster Virus
